# The association of circulating endocannabinoids with neuroimaging and blood biomarkers of neuro-injury

**DOI:** 10.1186/s13195-023-01301-x

**Published:** 2023-09-12

**Authors:** Shiraz Vered, Alexa S. Beiser, Liron Sulimani, Sharon Sznitman, Mitzi M. Gonzales, Hugo J. Aparicio, Charles DeCarli, Matthew R. Scott, Saptaparni Ghosh, Gil M. Lewitus, David Meiri, Sudha Seshadri, Galit Weinstein

**Affiliations:** 1 School of Public Health, University of Haifa, 199 Aba Khoushy Ave., Haifa, 3498838 Israel; 2 Department of Neurology, Boston University Chobanian and Avedisian School of Medicine, Boston, MA 02118 USA; 3 Department of Biostatistics, Boston University School of Public Health, Boston, MA 02118 USA; 4 The Framingham Study, Framingham, MA 01702 USA; 5 The Kleifeld Laboratory, Department of Biology, Technion-Israel Institute of Technology, 3200003 Haifa, Israel; 6 Glenn Biggs Institute for Alzheimer’s and Neurodegenerative Diseases, University of Texas Health Sciences Center, San Antonio, TX 78229 USA; 7 Department of Neurology, University of California at Davis, Sacramento, CA 95816 USA; 8 The Laboratory of Cancer Biology and Cannabinoid Research, Department of Biology, Technion-Israel Institute of Technology, 3200003 Haifa, Israel

**Keywords:** Endocannabinoids, Brain MRI, Blood biomarkers, Sex, Brain aging, Neuro-injury, Neurodegeneration

## Abstract

**Background:**

Preclinical studies highlight the importance of endogenous cannabinoids (endocannabinoids; eCBs) in neurodegeneration. Yet, prior observational studies focused on limited outcome measures and assessed only few eCB compounds while largely ignoring the complexity of the eCB system. We examined the associations of multiple circulating eCBs and eCB-like molecules with early markers of neurodegeneration and neuro-injury and tested for effect modification by sex.

**Methods:**

This exploratory cross-sectional study included a random sample of 237 dementia-free older participants from the Framingham Heart Study Offspring cohort who attended examination cycle 9 (2011–2014), were 65 years or older, and cognitively healthy. Forty-four eCB compounds were quantified in serum, via liquid chromatography high-resolution mass spectrometry. Linear regression models were used to examine the associations of eCB levels with brain MRI measures (i.e., total cerebral brain volume, gray matter volume, hippocampal volume, and white matter hyperintensities volume) and blood biomarkers of Alzheimer’s disease and neuro-injury (i.e., total tau, neurofilament light, glial fibrillary acidic protein and Ubiquitin C-terminal hydrolase L1). All models were adjusted for potential confounders and effect modification by sex was examined.

**Results:**

Participants mean age was 73.3 ± 6.2 years, and 40% were men. After adjustment for potential confounders and correction for multiple comparisons, no statistically significant associations were observed between eCB levels and the study outcomes. However, we identified multiple sex-specific associations between eCB levels and the various study outcomes. For example, high linoleoyl ethanolamide (LEA) levels were related to decreased hippocampal volume among men and to increased hippocampal volume among women (*β* ± SE =  − 0.12 ± 0.06, *p* = 0.034 and *β* ± SE = 0.08 ± 0.04, *p* = 0.026, respectively).

**Conclusions:**

Circulating eCBs may play a role in neuro-injury and may explain sex differences in susceptibility to accelerated brain aging. Particularly, our results highlight the possible involvement of eCBs from the N-acyl amino acids and fatty acid ethanolamide classes and suggest specific novel fatty acid compounds that may be implicated in brain aging. Furthermore, investigation of the eCBs contribution to neurodegenerative disease such as Alzheimer’s disease in humans is warranted, especially with prospective study designs and among diverse populations, including premenopausal women.

**Supplementary Information:**

The online version contains supplementary material available at 10.1186/s13195-023-01301-x.

## Introduction

Endocannabinoids (i.e.**,** endogenously produced cannabinoids; eCBs) are small lipophilic signaling molecules discovered following the recognition that the existence of cannabinoid receptor types 1 (CB1) and 2 (CB2) indicates the presence of endogenous ligands [1]. The “classic,” best-studied eCBs are 2-arachidonoyl (2-AG) and arachidonoyl ethanolamide (AEA), which bind directly to CB1 and CB2 receptors [2]. Yet, recent investigations discovered long-chain fatty acid amides and esters that often share biosynthetic or degradative enzymes with 2-AG and AEA and can thus considered to be part of the expanded eCB system (e.g., endocannabinoidome) [3]. Accumulating evidence highlights the essential role of the eCB system in multiple physiological functions such as energy homeostasis, food intake, sleep patterns, mood, and stress [4, 5]. In addition, the eCB signaling is implicated in numerous pathophysiological processes, including respiratory, mental, cardiovascular, and cerebrovascular diseases [6]. Hence, modulation of eCBs levels and their signaling offers promising therapeutic interventions [7].

Special attention has recently been raised to the possible implication of the eCB system in neurodegeneration processes and Alzheimer’s disease (AD) pathogenesis [8]. CB1 receptors are abundant in the excitatory and inhibitory pre-synaptic terminals, where they facilitate the inhibition of neurotransmitter release and regulate important brain functions including memory and cognition [9]. CB2 receptors are present mainly in immune system cells, but their expression is upregulated under specific conditions of neuroinflammation [10]. Activation of these receptors may affect the brain through multiple biological mechanisms, including inhibition of pre-synaptic neurotransmitter release and neuroinflammation, and reduction of tau phosphorylation, oxidative damage, and amyloid beta neurotoxicity [8, 11]. Furthermore, other “non-classic” eCB receptors such as the orphan receptors GPR3, GPR6, and GPR12 are implicated in neurodegenerative disorders [12]. Specifically, GPR3 was shown to modulate amyloid-beta generation and is considered a potential target for Alzheimer’s treatment [13]. A wealth of data also shows that the eCB system changes markedly with age and may be implicated in brain aging through various biological mechanisms [14].

Despite the plethora of evidence from animal models and human post-mortem brain samples, observational studies assessing the link of eCBs levels with neurodegeneration and AD biomarkers are scarce [8]. Of note, most panels used for novel system biology approaches (e.g., metabolomics, lipidomics) in AD research do not include eCB compounds [15]. Furthermore, the existing observational studies that focus on eCBs are limited by small sample sizes and by limited numbers of eCB compounds, thus ignoring the complexity of the eCB metabolome (eCBome) [16, 17].

In addition to increasing sample sizes and numbers of eCBs, it is important to consider the role of sex differences when moving forward with this research field [18, 19]. Evidence from preclinical studies suggests that the expression and activity of eCBs in the brain differ by sex [20]. Hence, it may be postulated that eCB levels may explain, at least partly, the sex differences observed in AD prevalence [21, 22] and during the preclinical phases of dementia and normal aging [23, 24].

In the current cross-sectional exploratory study, we measured the serum levels of 44 eCBs and eCB-like lipids in a sample of dementia-free older adults who participated in the Framingham Heart Study Offspring cohort. We related these eCB levels to brain MRI measures of neurodegeneration. We additionally assessed the link between eCBs and the following novel AD and neurodegeneration/neuro-injury blood biomarkers: total tau (t-tau), neurofilament light (NfL), glial fibrillary acidic protein (GFAP), and ubiquitin C-terminal hydrolase L1 (UCH-L1). We further assessed the possible role of sex as an effect-modifier.

## Methods

### Study sample

The Framingham Heart Study (FHS) is a single-site, longitudinal community-based cohort study that was initiated in 1948 [25]. Since its inception, three generations of participants have been enrolled. In 1971, the Offspring cohort was established, including 5124 children and children’s spouses of participants from the original cohort. To date, the Offspring cohort participants have completed up to ten quadrennial examinations. Further details on the design and selection criteria are described elsewhere [26]. For the current study, we included participants of the Offspring cohort who attended examination cycle 9 (2011–2014). Data from 250 participants were randomly selected from 736 eligible attendees, with the following inclusion criteria: (1) age 65 years or older; (2) without dementia, stroke, or other significant neurological diseases (e.g., head trauma, multiple sclerosis) at the time of the examination visit; (3) completed brain magnetic resonance imaging (MRI) after the clinical examination; and (4) had dementia follow-up. We excluded 13 individuals who had incident dementia or stroke diagnosed after the clinical but before the MRI examinations. Thus, the final sample for the current analysis included 237 individuals. Comparison of the main characteristics between study sample and offspring cohort participants who attended exam nine but who were not included in the study are presented in Supplementary Table 1. Data were obtained under a protocol approved by the institutional review board of the Boston University Medical Center, and written informed consent was obtained from all participants.

### Circulating endocannabinoid levels

Serum from fasting blood draws performed at examination nine were used to assay eCBs levels. Serum stored in the FHS laboratory at – 80 °C was shipped to the Laboratory of Cancer Biology and Cannabinoid Research (LCBCR), Faculty of Biology, Technion–Israel Institute of Technology, Haifa, Israel, in tubes of 0.2 mL on dry ice. The shipment also included 14 phantom specimens for assessment of measurement reliability. During shipment, the temperature was monitored using a temperature data logger. eCBs and eCB-like compounds were simultaneously extracted, identified, and quantified in serum, via liquid chromatography high-resolution mass spectrometry (LC/HRMS). Detailed description of the development and validation of the methods has been previously described [27]. In brief, 600 μL of the extraction solution (0.1% v/v acetic acid in a methanol to acetonitrile 1:1 mixture) spiked with 20 ng/mL deuterated internal standards (ISs) were added to 200 μL of serum samples. Samples were thoroughly vortexed and then centrifuged for 20 min at 4 °C for protein and cell precipitation. The supernatants were then extracted using Agela Cleanert C8 solid phase extraction (SPE) cartridges. eCBs in serum samples were analyzed using a Thermo Scientific UHPLC system coupled with a Q Exactive Focus Hybrid Quadrupole-Orbitrap MS (Thermo Scientific, Bremen, Germany). The chromatographic separation was carried out on a Halo C18 Fused Core column (2.7 μm, 150 mm × 2.1 mm i.d.). eCBs were expressed as ng/mL. Additionally identified eCBs with no commercially available analytical standards were semi-quantified according to the standard curve of a compound from the same lipid family. This method was validated for quantification of the eCBome in serum according to the US Food and Drug Administration guidelines over three consecutive days, for linearity, limit of quantitation, precision, accuracy, and stability [28]. In short, acceptable accuracies for the vast majority of eCBs ranged between 90 and 110% and had less than 15% repeatability and reproducibility values.

Overall, 58 eCBs were tested in the serum. Of them, 14 eCBs had > 90% missing values (due to either inability to detect an accurate measure or because the quantity was below detectable levels). Thus, 44 eCBs were analyzed for an association with the study outcomes. Ten eCBs that had at least 50% of values reaching below detectable levels were recoded as a binary variable with 0 = lowest detectable value and 1 = above detectable value. The other 34 eCBs were analyzed as continuous variables with normal distributions. Outliers defined as ± 4 standard deviation from the mean were excluded (< 2% of the values). A list of the eCB compounds assessed in the current study along with their classification and abbreviations is presented in Supplementary Table 2.

### Brain MRI measures

Attendees of the 9th clinical examination were invited to participate in the MRI study and undergo brain MRI. Brain MRI images were obtained on a Siemens Magnetom and a Phillips Achieva 3 Tesla scanners using T1-weighted coronal spoiled gradient-recalled echo acquisition and fluid-attenuated inversion recovery sequences with standard MRI parameters. All images were transferred to and processed by the University of California Davis Medical Center without knowledge of clinical information. Segmentation and quantification of brain volume measures were performed by automated procedures with quality control. Additional information on imaging methodology was previously described [29]. The current analysis included measures of total cerebral brain volume (TCBV), gray matter volume, hippocampal volume corrected for differences in head size by analyzing these variables as percentages of total cranial volume, and white matter hyperintensities volume analyzed as percentage of total brain volume [30]. In addition, white matter hyperintensities volumes were log-transformed to normalize the distribution.

### Quantification of AD and neuro-injury blood biomarkers

Circulating levels of NfL, GFAP, t-tau, and UCH-L1 were quantified in plasma using the Simoa Neurology 4-Plex Kit on a Simoa HD-1 Analyzer (Quanterix). Outliers (**± **4 standard deviations from the mean) were excluded, and all biomarkers were log-transformed to normalize their distributions.

### Statistical analysis

Continuous and categorical variables were summarized as means and standard deviations and as frequencies and percentages, respectively. Multiple linear regression models adjusted for age, age^2^, sex, and APOE genotype were used to examine the association of each eCB with MRI and blood biomarkers. Age squared was included to allow modeling of a quadratic age effect on the outcomes. Models of brain MRI outcomes further adjusted for time between blood draw and the MRI examination. For each model, significant results were considered as *p* value < 0.05 and additionally corrected for multiple testing using the Bonferroni method. Furthermore, we examined whether sex modified the associations between eCB levels and the study outcomes by including interaction terms of each eCB with sex in regard to the outcome measures. In cases where the eCB*sex interaction was significant (*p* value < 0.10 due to low power of such tests), we ran stratified analyses by sex. All statistical analyses were conducted using SAS version 9.4.

## Results

Sample characteristics overall and by sex are presented in Table 1. Of the 237 participants, 95 (40%) were men and the mean age was 73.3 ± 6.2 years. The mean time duration between the clinic examination (i.e., blood draw) and MRI assessments were 1.7 ± 0.9 years.

**Table 1:** Sample characteristics

Variables		Overall *N* = 237 (100%)	Women *N* = 142 (60%)	Men *N* = 95 (40%)
Age, years		73.3 ± 6.2	73.6 ± 6.6	72.8 ± 5.5
Education	No college	72 (30.4)	49 (34.5)	23 (24.2)
	Some college	75 (31.6)	52 (36.6)	23 (24.2)
	College graduate	90 (38.0)	41 (28.9)	49 (51.6)
Current smoking		8 (3.4)	5 (3.5)	3 (3.2)
Body mass index (kg/m^2^) ≥ 30		64 (27.0)	40 (28.2)	24 (25.3)
APOE4 positive		46 (19.8)	26 (18.8)	20 (21.3)
Time between blood draw and cognitive assessment, years		1.6 ± 1.0	1.6 ± 1.0	1.7 ± 1.0
Global cognition performance^a^		− 0.26 ± 1.1	− 0.14 ± 1.1	− 0.44 ± 1.0
**Brain MRI measures**				
Time between blood draw and MRI, years		1.7 ± 0.9	1.7 ± 1.0	1.7 ± 0.9
Total cerebral brain volume, % TCV		74.1 ± 2.1	74.2 ± 2.1	74.0 ± 2.2
Gray matter volume, % TCV		40.0 ± 1.9	40.4 ± 2.0	39.3 ± 1.7
Hippocampal volume, % TCV		0.52 ± 0.05	0.52 ± 0.04	0.50 ± 0.04
White matter hyperintensities, % TCV		0.3 [0.1–0.5]	0.3 [0.1–0.6]	0.3 [0.1–0.4]
**Blood biomarkers**				
Neurofilament light, pg/mL		20.7 [13.9–28.0]	21.5 [14.0–27.5]	18.8 [13.9–28.8]
Glial fibrillary acidic protein, pg/mL		193.6 [148.4–269.4]	209.2 [152.4–278.9]	182.8 [131.1–249.1]
Total tau, pg/mL		1.7 [1.2–2.4]	1.9 [1.3–2.5]	1.4 [1.0–1.9]
UCH-L1, pg/mL		25.4 [20.7–35.9]	25.8 [20.7–35.8]	25.3 [20.6–37.1]

Continuous trait values are reported as mean ± SD or median [IQR] and dichotomous trait values are reported as number (percent)

*Abbreviations*: *APOE4* apolipoprotein ɛ4, *MRI* magnetic resonance imaging, *TCV* total cranial volume, *UCH-L1* ubiquitin carboxyl-terminal hydrolase L1

^a^Global cognitive performance score was constructed as the first unrotated component in principal component analysis based on the following cognitive tasks: Logical Memory (immediate and delayed), Visual Reproductions (immediate and delayed), Psychogeriatric Assessment Score (immediate and delayed), Similarities, Hooper Visual Organization Test and Trails making B

**Fig. 1:**
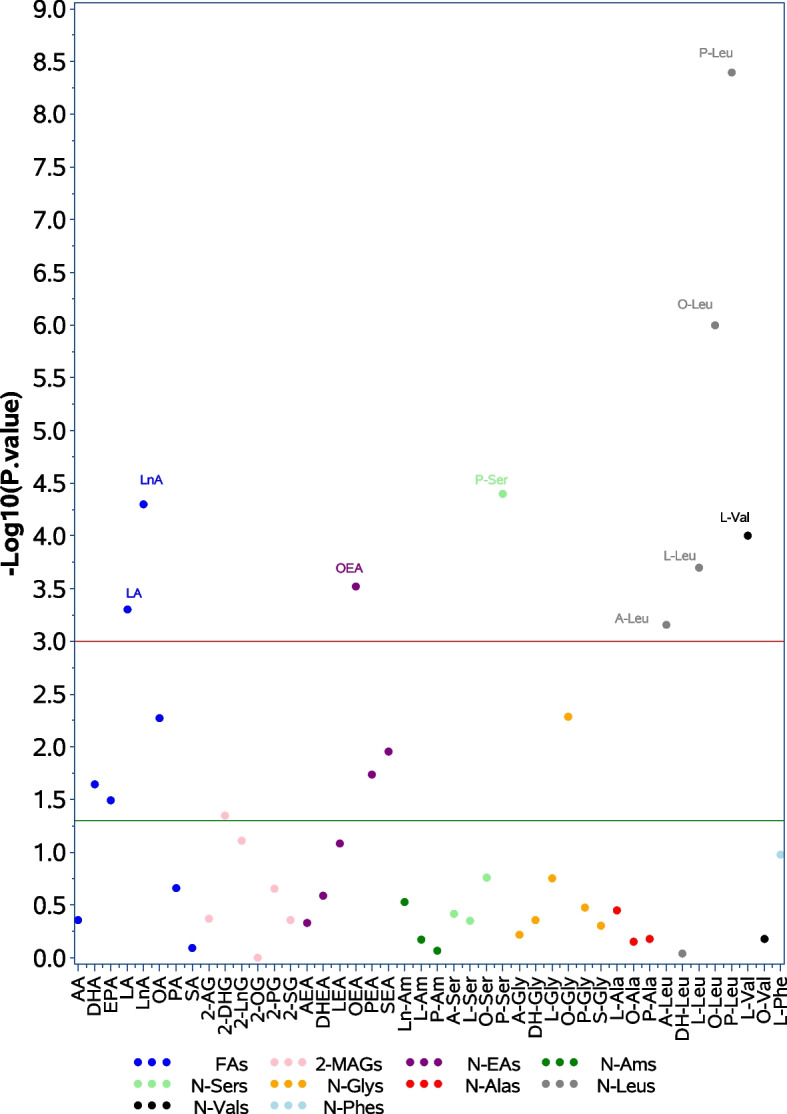
The association of sex with endocannabinoid levels. Green line indicates *p* value of 0.05, red line indicates *p* value after Bonferroni correction for multiple comparisons. Endocannabinoids are colored by families. For abbreviations, see Supplementary Table 2

The associations of each eCB levels with sex, brain MRI, and blood biomarkers are presented in Fig. 1, Supplementary Fig. 1, and Supplementary Fig. 2, respectively. The 44 studied eCBs are displayed along the *x*-axis according to the lipid classes (denoted by different colors). The value on the *y*-axis represents the − log10 of the *p* value. *p* values for each association are presented on the eCBs scale. The significance level of 0.05 is plotted as the green line, and the significance level after Bonferroni correction for multiple testing (0.05 divided by 44 analyses =  ~ 0.001) is plotted as the red line.

### The association of eCBs levels with sex

Figure 1 shows the relationship between sex and eCB levels. The nine following eCBs were different in men compared to women after considering multiple testing: LA, LnA, and OEA were higher in women compared to men, while L-Leu, O-Leu, P-Leu, A-Leu, P-Ser, and L-Val were higher in men compared to women (Supplementary Table 3). Another seven eCBs differed by sex using a nominal level of significance (< 0.05): DHA, EPA, OA, PEA, SEA, 2-DHG, and O-Gly were all higher in women compared to men.

### The associations of eCBs levels with MRI measures and blood biomarkers in the total sample

Overall, none of the eCBs were significantly associated with brain MRI measures after correction for multiple testing (Supplementary Fig. 1 and Supplementary Table 4). Similarly, Supplementary Fig. 2 and Supplementary Table 5 show that none of the eCBs reached statistical significance in relation to plasma levels of NfL, GFAP, t-tau, and UCH-L1 after correction for multiple testing.

### The associations of eCBs levels with MRI measures and blood biomarkers by sex

Significant interactions between eCBs and sex were observed in relation to all the study outcomes (Supplementary Fig. 3).

**Fig. 2:**
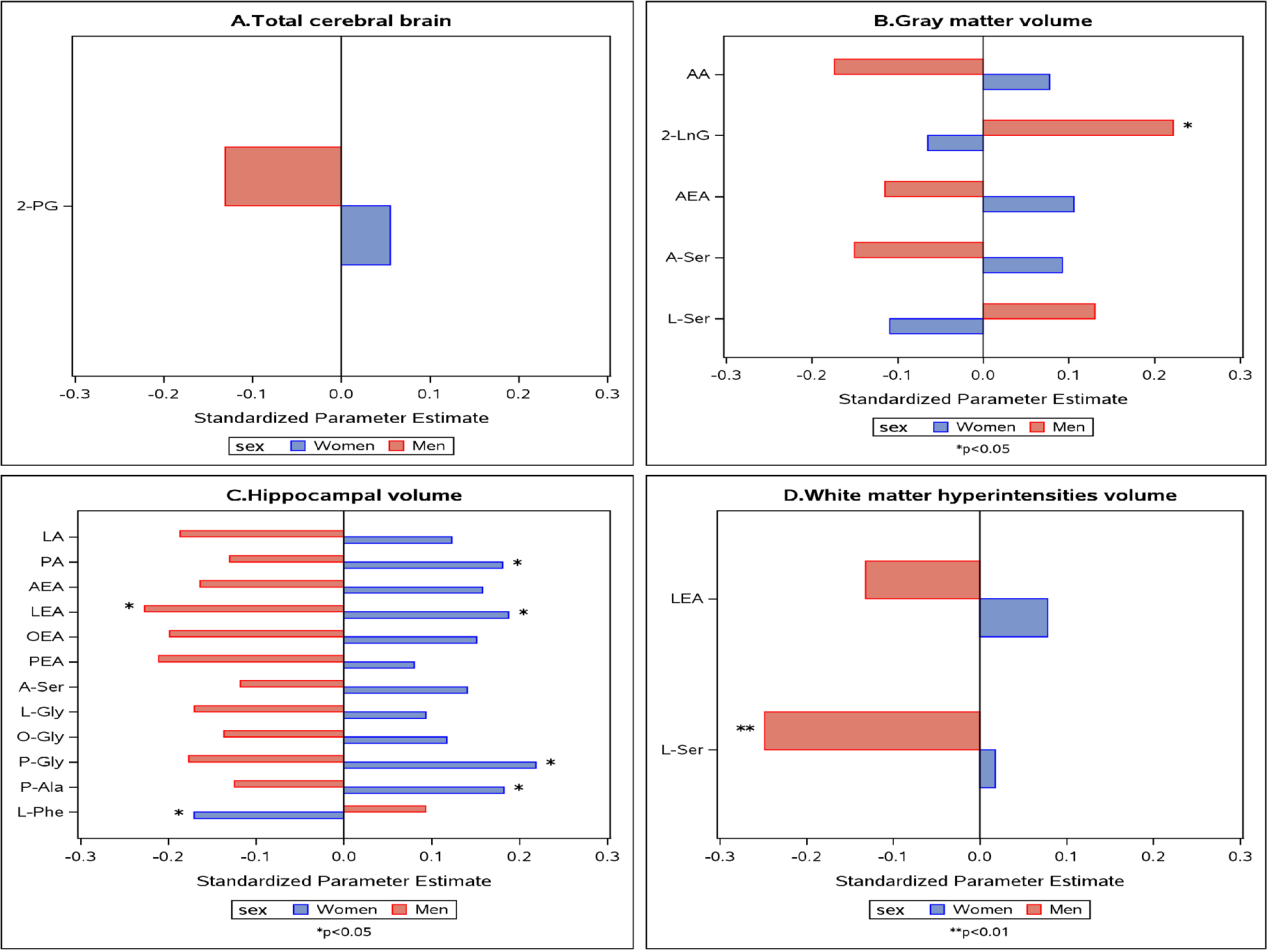
The associations between endocannabinoids and MRI measures by sex. Only significant endocannabinoid*sex interactions (*p* < 0.1) are shown. Models adjust for age, age^2^, APOE genotype, and time between blood draw and MRI. *X-axis shows standardized parameter estimates. For abbreviations, see Supplementary Table 2

**Fig. 3:**
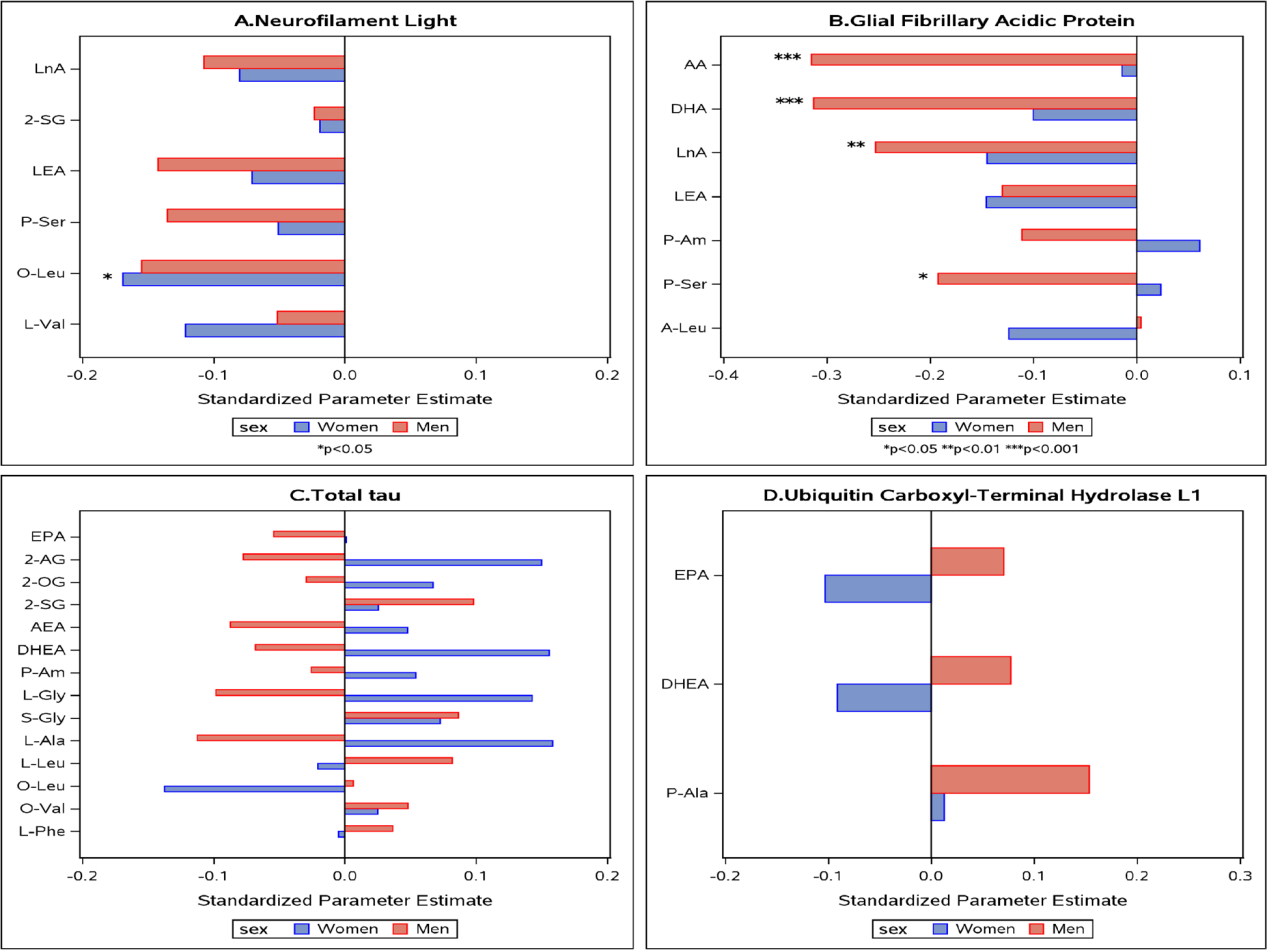
The associations between endocannabinoids and AD biomarkers by sex. Only significant endocannabinoid*sex interactions (*p* < 0.1) are shown. Models adjust for age, age^2^, APOE genotype. *X-axis shows standardized parameter estimates. For abbreviations, see Supplementary Table 2

Figure 2 and Supplementary Table 4 show that with respect to brain MRI measures, all the interactions between eCB levels and sex were such that men and women showed opposite direction of associations. Similarly, the associations of eCB levels with t-tau and UCH-L1 were mostly in opposite direction of associations (Fig. 3 and Supplementary Table 5). Effect modification by sex was additionally observed for NfL and GFAP. For O-Leu, higher levels were associated with lower NfL levels in both sexes and significantly in women, and higher levels of AA, DHA, LnA, and P-Ser were related to lower GFAP only in men (Fig. 3 and Supplementary Table 5).

A large proportion of significant associations found in our study were among eCBs from the N-acyl amino acids family. For example, increased levels of P-Gly and P-Ala and decreased levels of L-Phe were significantly related to larger hippocampal volume in women (*β* = 0.03 ± 0.01; *p* = 0.012, *β* = 0.23 ± 0.11; *p* = 0.038 and *β* =  − 0.02 ± 0.01; *p* = 0.039, respectively). In men, higher levels of L-Ser were related to smaller white matter hyperintensities volume (*β* =  − 7.59 ± 2.87; *p* = 0.010), and higher levels of P-Ser were related to decreased GFAP levels (*β* =  − 0.68 ± 0.33; *p* = 0.043). Additionally, high levels of O-Leu were significantly related to less NfL in women (*β* =  − 0.63 ± 0.28; *p* = 0.030).

Several significant relationships were observed between fatty acid (N-acyl) ethanolamide eCBs and the study outcomes. Among those are LEA, which was positively associated with hippocampal volume in women and negatively in men (*β* = 0.08 ± 0.04; *p* = 0.0.026 and *β* =  − 0.12 ± 0.06; *p* = 0.034, respectively).

Lastly, several associations of fatty acid eCBs were observed. For example, elevated PA levels were related to larger hippocampal volume in women (*β* = 0.00 ± 0.00; *p* = 0.035), and elevated AA, DHA, and LnA were related to lower GFAP levels in men (*β* =  − 0.002 ± 0.001; *p* < 0.001 and *β* =  − 0.002 ± 0.001; *p* < 0.001 and *β* =  − 0.001 ± 0.000; *p* = 0.007, respectively). A summary of the study findings can be found in Supplementary Table 6.

## Discussion

To the best of our knowledge, the current study is the first to assess the link of the eCBome with neuro-injury biomarkers. We were able to detect 44 eCB compounds in serum samples from 237 cognitively healthy adults aged ≥ 65 years, who participated in examination nine of the FHS Offspring cohort. We found that over 20% (nine out of 44) of the eCBs were associated with sex after correction for multiple comparisons. Overall, no associations of eCB levels with brain MRI measures and blood biomarkers were observed after correction for multiple testing. However, we identified robust interactions of eCB levels with sex. Sex stratification revealed a trend toward opposite direction of the relationship between the eCB levels and brain measures in men compared to women. When men and women were assessed separately, several novel associations were identified between eCB compounds and the study outcomes.

Although sex-specific levels of eCB levels have rarely been reported in the literature, sex differences in the human metabolome are known [31]. In one study, 507 metabolites were analyzed in 1756 older adults (women were mainly menopausal). Gender-specific differences were observed in more than one third of the tested metabolites (180 out of 507) [31]. Specifically, we found higher OEA levels in our sample of women in menopausal age compared to men, which may be related to increasing levels of OEA in post-menopausal state [32].

In addition to our observation of sex differences in the levels of eCB molecules, our results point to a potentially strong interaction between sex and the eCB signaling with regard to early neurodegeneration and neuro injury markers. In line with our findings, a study from the Alzheimer’s Disease Neuroimaging Initiative (ADNI) cohorts identified substantial differences between men and women in their associations of blood metabolome with A-T-N biomarkers [33]. Sex-specific differences were observed mainly in metabolites responsible for energy metabolism and homeostasis, processes that are regulated by eCBs [4, 5]. Sex-specific differences has been shown in the expression and affinity of cannabis receptors in animals [34] and human brains [35]. Hence, cannabis signaling through these receptors may differentially affect brain health in men and women, as demonstrated by our findings. Furthermore, emerging evidence suggests that the eCB and estrogen systems interact to differentially affect brain development, learning, and memory [36].

The significant associations of eCBs levels with the study outcomes, in either men or women, were observed across different families of eCB compounds. For example, we show that sex modified several associations of fatty acid (N-acyl) ethanolamides, particularly with hippocampal volume (AEA, LEA, OEA, and PEA) and white matter hyperintensities (LEA), all showed an opposite direction of association comparing men to women. Fatty acid amides (grouped largely into fatty acid ethanolamides and fatty acid primary amides) are considered bioactive signaling molecules with a variety of physiological functions, including regulation of sleep and food intake [37], but their relationship with neurodegeneration is unclear. The levels of these molecules are regulated by fatty acid amide hydrolase (FAAH) and monoacylglycerol lipase (MAGL) [38, 39]. FAAH and MAGL inhibitors possess several neuroprotective effects and are therefore considered promising molecules in the prevention of cognitive decline and AD pathogenesis [38, 40]. Moreover, in line with our findings, evidence suggests that the effects of FAAH on the brain may be affected by sex hormones. For example, it has been shown that estrogen affects emotional behavior in rats by modulating eCB activity, mainly through inhibition of FAAH [41]. Our findings suggest that increased levels of fatty acid amides can be related to either improved or poorer brain health, depending on the specific molecule and sex. For example, we found that in men, elevated LEA levels are significantly related to smaller hippocampal volume, with an opposite trend in women. This heterogeneity of findings may also explain the inconsistency in the literature, where some studies suggest an overexpression [42] and others diminished expression [43] of FAAH activity in AD brains. Furthermore, our findings suggest that using FAAH/MAGL inhibitors for the prevention of cognitive impairment should be sex specific. Of note, eCB belonging to the fatty acid primary amides class (e.g., Ln-Am, L-Am, and P-Am) was not related to the outcomes in our study. This stands in contrast to a recent study that explored a panel of 883 plasma metabolites demonstrating a significant relationship between high levels of L-Am and lower hippocampal volume [44]. This inconsistency may be due to differences in the study sample, which in the prior research included not only cognitively healthy participants as in the current sample but also participants with mild cognitive impairment and AD that may have higher L-Am levels [44].

It is well recognized that fatty acids are implicated in brain development and function [45]. Particularly, evidence suggests that dietary intake of DHA may reduce AD risk [46]. Furthermore, a recent study among cognitively healthy participants of the Framingham Study 3rd Generation demonstrated an association between high DHA concentrations in red blood cells with larger hippocampal volume and improved abstract reasoning [47]. Our current study expands this knowledge by showing that higher serum levels of eCBs from the fatty acid family are related to decreased blood biomarkers of neurodegeneration and neuro-injury. Particularly, elevated DHA and AA levels were related to lower GFAP, and these associations were present in men only. We additionally show a robust sex interaction of fatty acid eCBs with hippocampal volume. One of these fatty acids is PA, a saturated fatty acid with essential functions in the brain. Yet, research suggests that excessive amounts of PA may increase the risk for AD and other neurodegenerative disorders [42]. In this context, our findings concur with the detrimental effects by demonstrating lower hippocampal volume in men; however, in our sample of post-menopause women, elevated PA levels were significantly related to larger hippocampal volume.

Our findings stress the possible key role of various N-acyl amino acids in neuro-injury processes. For example, we show that among men, several amino acids were significantly associated with lower levels of GFAP. Despite a growing interest in these molecules, little is currently known regarding their role in biological processes and pathophysiology [48, 49]. Further exploration of this group of compounds in the context of brain aging and neuro-injury is warranted, particularly since N-acyl amino acids, similarly to fatty acids, are essential diet elements, and their levels may be modulated by food.

The mechanisms underlying the observed associations of eCBs with neuro-injury biomarkers in our study were explored extensively in prior preclinical research. eCBs are neurotransmitters that regulate important brain functions including memory and cognition [50]. After being synthesized on demand in response to neuronal membrane depolarization or immune cell activation, the eCBs are released from post-synaptic membranes onto presynaptic terminals, where they can suppress inhibitory and excitatory signaling and control synaptic plasticity [51]. Furthermore, the eCB system has a pivotal role in brain inflammation by regulating microglial activity in healthy and neurodegenerative conditions [52]. Lastly, eCBs are known to exert neuroprotective effects through multiple pathways, including through the reduction of tau phosphorylation, oxidative damage, and amyloid beta neurotoxicity [53], and are implicated in the cellular and molecular pathways of brain aging and pathological neurodegeneration [14]. However, it should be noted that information on eCB mechanisms is derived primarily from investigations of the classic eCB rather than the expanded set of compounds.

Several limitations of our study should be acknowledged. (1) Due to the study’s cross-sectional design, we cannot infer a temporal relationship between eCBs levels and the study outcomes. (2) Circulating eCBs levels may not reflect brain signaling [27]. Yet, eCBs can cross the blood brain barrier and their circulating levels were linked with AD-related factors such as cardiovascular diseases, obesity, and depression [5, 54]. (3) Although we used the optimal laboratory practice (e.g., plasma frozen without delay, no repeated freeze/thaw cycles, blood draw in resting state after fasting), it is important to note that eCBs have limited stability. Yet, it should be noted that any misclassification of eCBs levels is expected to be non-differential and may therefore result in attenuation of the real association. (4) During the mean time period of 1.7 years that passed between the blood sample collection and the brain MRI examination, changes in circulating eCB levels may have occurred, and little is known on the changes in eCBs levels over time in humans. (5) Our external validity is restricted to predominantly European ancestry, from one geographic area and of a relatively high socioeconomic status. In particular, due to the inclusion of individuals aged 65 years and above, the women in the current sample were at post-menopausal status. (6) Despite the large sample size compared to previous literature, statistical power of our study was still limited. Therefore, we consider our findings preliminary and encourage validation of our findings in larger and diverse samples. (7) Although we adjusted for the main relevant covariates, residual confounding could also affect our results. Particularly, the inclusion of medication intake as a possible confounder should be considered in future research.

## Conclusion

We identified a wide range of sex-specific associations of novel eCB compounds with neuro-injury biomarkers among older adults. These findings suggest putative molecular pathways underlying brain aging and pathological neurodegeneration and highlight mechanisms behind sex differences in susceptibility to neurodegenerative diseases. Further investigation of the eCBs contribution to neurodegeneration in humans is warranted, particularly with prospective study designs, and among diverse populations, including among younger individuals, pre-menopausal women, and older adults with AD and related neurodegenerative disorders. A better understanding of the complex eCB system and its interaction with sex may lead to starting points for future modulation of the eCB system through already available natural or synthetic cannabinoids as therapeutic strategies and to the development of sex-specific personalized health care in neurodegenerative disorders.

### Supplementary Information

**Additional file 1:** **Supplementary Figure 1.** Associations between endocannabinoid levels and MRI measures. Green line indicates *p* value of 0.05, red line indicates *p* value after Bonferroni correction for multiple comparisons. Models adjust for age, age^2^, sex, APOE genotype and time between blood draw and MRI. Endocannabinoids are colored by families. For abbreviations see Supplementary Table 2.

**Additional file 2:** **Supplementary Figure 2.** Associations of endocannabinoid levels with AD and neuro-injury blood biomarkers. Green line indicates *p* value of 0.05, red line indicates *p* value after Bonferroni correction for multiple comparisons. Models adjust for age, age^2^, sex, APOE genotype. Endocannabinoids are colored by families. For abbreviations see Supplementary Table 2.

**Additional file 3:** **Supplementary Figure 3.** Interactions (*p *< 0.1) between endocannabinoids and sex. Models adjust for age, age^2^, APOE genotype and models of MRI outcomes also adjusted for time between blood draw and MRI. A=Total cerebral brain, B=Gray matter, C=Hippocampus, D=White matter hyperintensities, E=Neurofilament Light, F=Glial Fibrillary Acidic Protein, G=Total Tau, H=Ubiquitin Carboxyl-Terminal Hydrolase L1 For abbreviations see Supplementary Table 2.

**Additional file 4:** **Supplementary Table 1 .** Characteristics of the study sample and participants not included in the analyses.

**Additional file 5:** **Supplementary Table 2.** List of endocannabinoids by family and abbreviations.

**Additional file 6:** **Supplementary Table 3.** Separate analysis results (T test and chi-square) for each eCBs comparing between women and men.

**Additional file 7:** **Supplementary Table 4.** Separate regression analysis results for each eCBs in relation to brain MRI measures. Showing crude models, adjusted models, models with interaction and stratified models by sex.

**Additional file 8:** **Supplementary Table 5.** Separate regression analysis results for each eCBs in relation to AD and neuro-injury biomarkers. Showing crude models, adjusted models, models with interaction and stratified models by sex.

**Additional file 9:** **Supplementary Table 6.** Summary of the study results.

## Data Availability

Deidentified data are available through formal data request application procedures by qualified investigators. More information is presented in framinghamheartstudy.org or biolincc.nhlbi.nih.gov/studies/framcohort/.
