# Supplementary material for: The association of circulating endocannabinoids with neuroimaging and blood biomarkers of neuro-injury
Source: Alzheimers Res Ther. 2023 Sep 12;15:154. doi: 10.1186/s13195-023-01301-x (PMC10496329; doi:10.1186/s13195-023-01301-x)
Supplement: Supplementary file 1 — Additional file 1: Supplementary Figure 1. Associations between endocannabinoid levels and MRI measures. Green line indicates p value of 0.05, red line indicates p value after Bonferroni correction for multiple comparisons. Models adjust for age, age2, sex, APOE genotype and time between blood draw and MRI. Endocannabinoids are colored by families. For abbreviations see Supplementary Table 2. [file 13195_2023_1301_MOESM1_ESM.pdf]

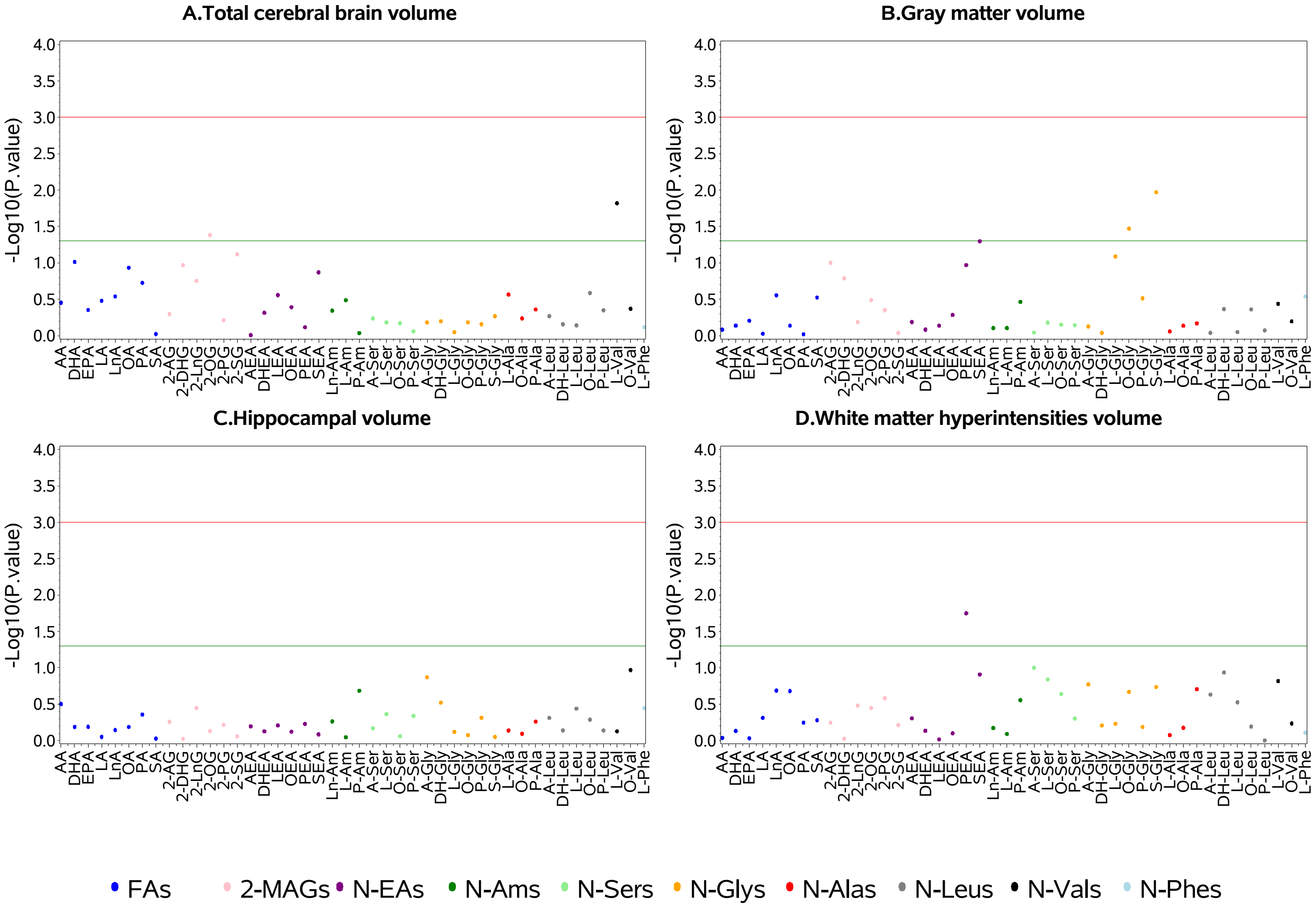

A.Total cerebral brain volume
B.Gray matter volume
C.Hippocampal volume
D.White matter hyperintensities volume
-Log10(P.value)
0.0 0.5 1.0 1.5 2.0 2.5 3.0 3.5 4.0
AA DHA EPA LA LnA OA PA SA 2-AG 2-DHG 2-LnG 2-OG 2-PG 2-SG AEA DHEA LEA OEA PEA SEA Ln-Am L-Am P-Am A-Ser L-Ser O-Ser P-Ser A-Gly DH-Gly L-Gly O-Gly P-Gly S-Gly L-Ala O-Ala P-Ala A-Leu DH-Leu L-Leu O-Leu P-Leu L-Val O-Val L-Phe
FAs
2-MAGs
N-EAs
N-Ams
N-Sers
N-Glys
N-Alas
N-Leus
N-Vals
N-Phes
