# Supplementary material for: The association of circulating endocannabinoids with neuroimaging and blood biomarkers of neuro-injury
Source: Alzheimers Res Ther. 2023 Sep 12;15:154. doi: 10.1186/s13195-023-01301-x (PMC10496329; doi:10.1186/s13195-023-01301-x)
Supplement: Supplementary file 2 — Additional file 2: Supplementary Figure 2. Associations of endocannabinoid levels with AD and neuro-injury blood biomarkers. Green line indicates p value of 0.05, red line indicates p value after Bonferroni correction for multiple comparisons. Models adjust for age, age2, sex, APOE genotype. Endocannabinoids are colored by families. For abbreviations see Supplementary Table 2. [file 13195_2023_1301_MOESM2_ESM.pdf]

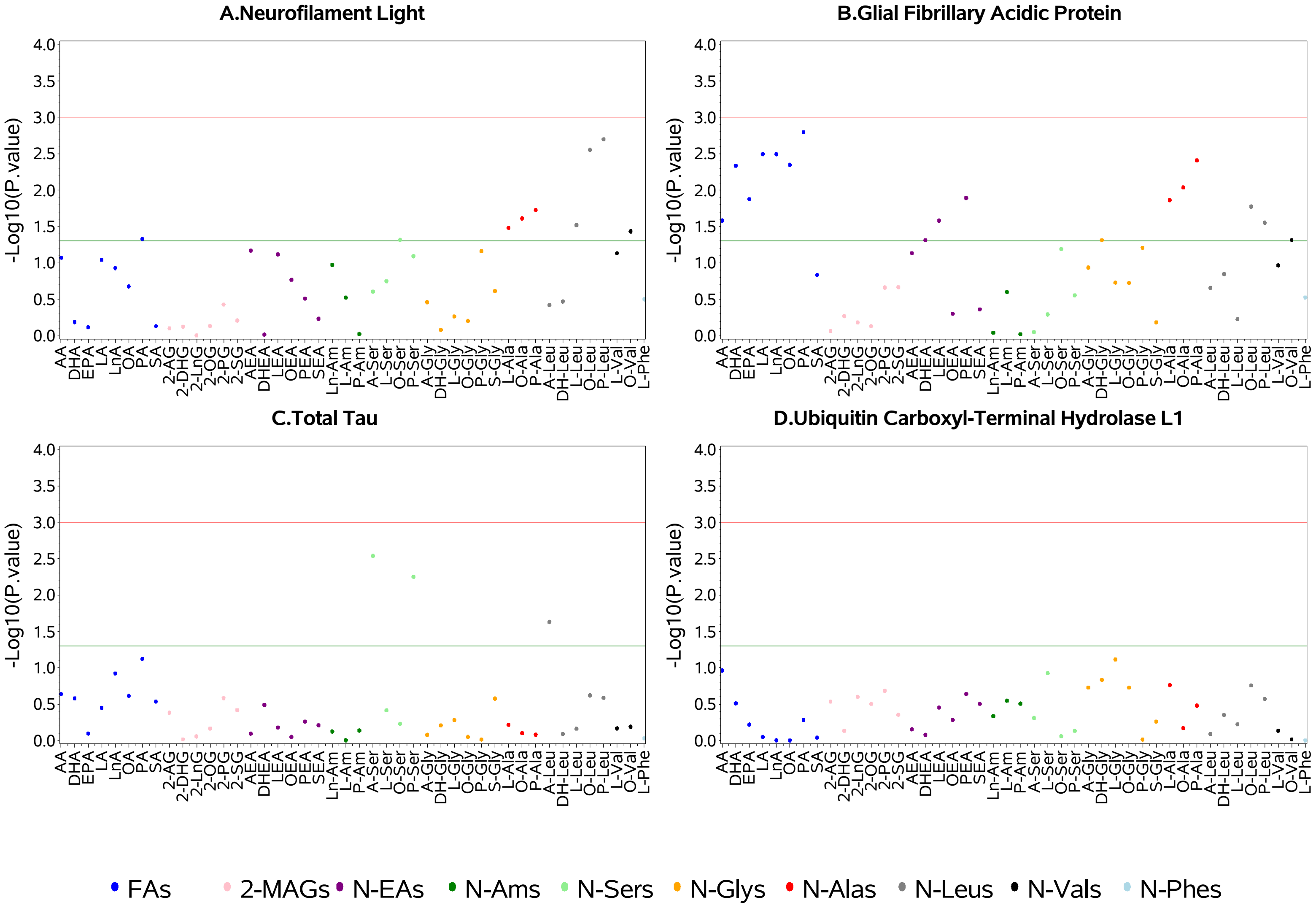
A.Neurofilament Light
B.Glial Fibrillary Acidic Protein
C.Total Tau
D.Ubiquitin Carboxyl-Terminal Hydrolase L1
-Log10(P.value)
AA DHA EPA LA LnA OA PA SA 2-AG 2-DHG 2-LnG 2-OG 2-PG 2-SG AEA DHEA LEA OEA PEA SEA Ln-Am L-Am P-Am A-Ser L-Ser O-Ser P-Ser A-Gly DH-Gly L-Gly O-Gly P-Gly S-Gly L-Ala O-Ala P-Ala A-Leu DH-Leu L-Leu O-Leu P-Leu L-Val O-Val L-Phe
FAs
2-MAGs
N-EAs
N-Ams
N-Sers
N-Glys
N-Alas
N-Leus
N-Vals
N-Phes
