# Supplementary figures and images for: The association of circulating endocannabinoids with neuroimaging and blood biomarkers of neuro-injury
Source: Alzheimers Res Ther. 2023 Sep 12;15:154. doi: 10.1186/s13195-023-01301-x (PMC10496329; doi:10.1186/s13195-023-01301-x)

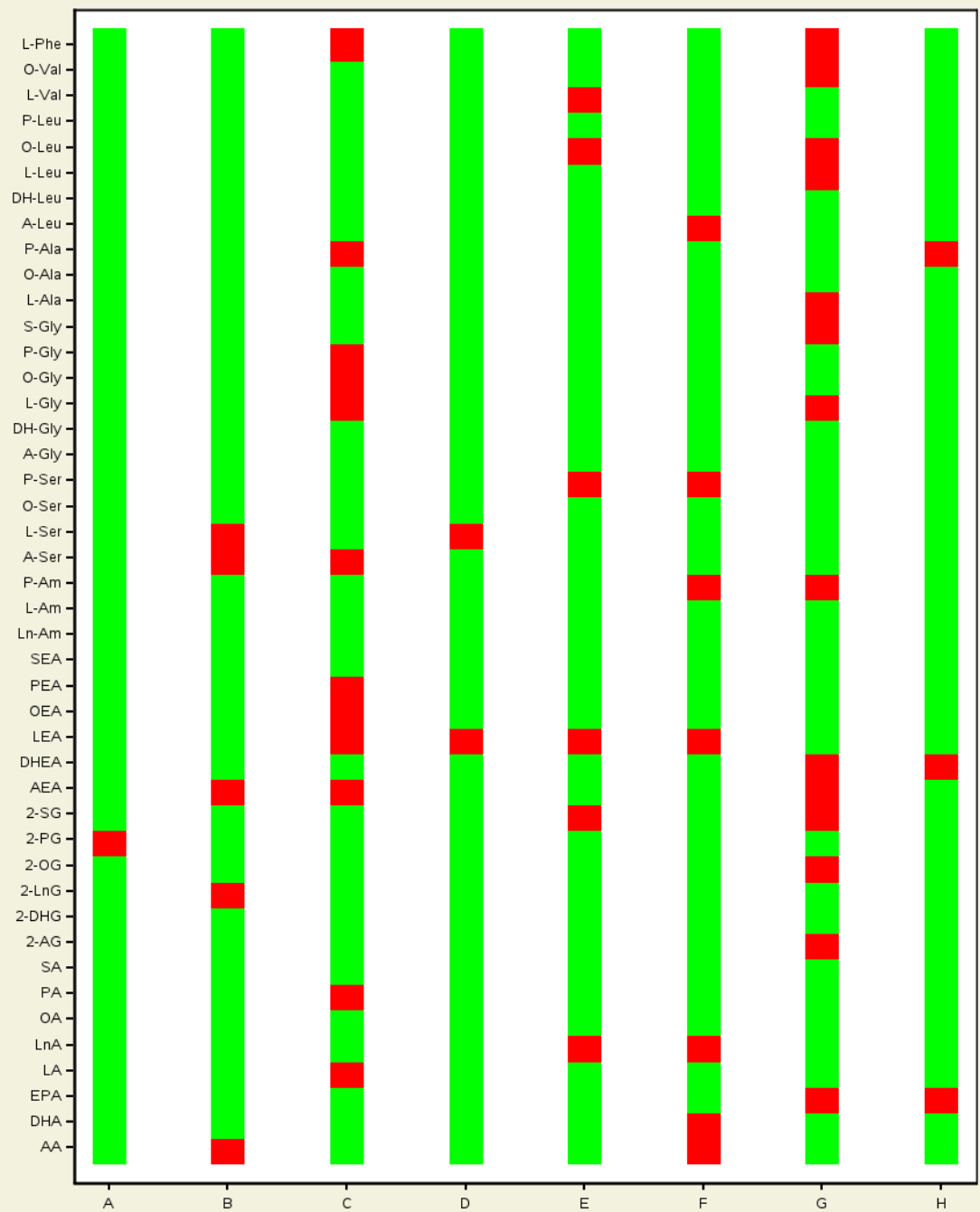

eCB\*sex interactions

Green  $p > 0.1$

Red  $p < 0.1$

Supplement: Supplementary file 3 — Additional file 3: Supplementary Figure 3. Interactions (p < 0.1) between endocannabinoids and sex. Models adjust for age, age2, APOE genotype and models of MRI outcomes also adjusted for time between blood draw and MRI. A=Total cerebral brain, B=Gray matter, C=Hippocampus, D=White matter hyperintensities, E=Neurofilament Light, F=Glial Fibrillary Acidic Protein, G=Total Tau, H=Ubiquitin Carboxyl-Terminal Hydrolase L1 For abbreviations see Supplementary Table 2. [file 13195_2023_1301_MOESM3_ESM.pdf]
