# Supplementary material for: The association of circulating endocannabinoids with neuroimaging and blood biomarkers of neuro-injury
Source: Alzheimers Res Ther. 2023 Sep 12;15:154. doi: 10.1186/s13195-023-01301-x (PMC10496329; doi:10.1186/s13195-023-01301-x)
Supplement: Supplementary file 4 — Additional file 4: Supplementary Table 1 . Characteristics of the study sample and participants not included in the analyses. [file 13195_2023_1301_MOESM4_ESM.docx]

**Supplementary Table 1.** Characteristics of the study sample and participants not included in the analyses

| **Variables** | | **Included random sample**  **N=237** | **Not included ^a^**  **N=509** |
| --- | --- | --- | --- |
| Age, y | | 73.3 ±6.2 | 72.8±5.8 |
| Sex (Men) | | 95 (40.1) | 241 (47.4) |
| Education | No college degree | 72 (30.4) | 133 (26.1) |
|  | Some college | 75 (31.7) | 149 (29.3) |
|  | College graduate | 90 (38.0) | 227 (44.6) |
| Current smoking | | 8 (3.4) | 16 (3.2) |
| Body Mass Index (kg/m^2^) ≥ 30 | | 64 (27.0) | 157 (31.3) |
| APOE4 positive | | 46 (19.8) | 118 (23.9) |

Abbreviations: APOE4=apolipoprotein ɛ4;

Continuous values are reported as mean ±SD and dichotomous values are reported as N (%).

^a^ Offspring cohort participants who attended exam nine but were not included in the study
