# Supplementary material for: The association of circulating endocannabinoids with neuroimaging and blood biomarkers of neuro-injury
Source: Alzheimers Res Ther. 2023 Sep 12;15:154. doi: 10.1186/s13195-023-01301-x (PMC10496329; doi:10.1186/s13195-023-01301-x)
Supplement: Supplementary file 5 — Additional file 5: Supplementary Table 2. List of endocannabinoids by family and abbreviations. [file 13195_2023_1301_MOESM5_ESM.docx]

**Supplementary Table 5**

| General family | Lipid class | Endocannabinoids |
| --- | --- | --- |
| Fatty Acids | Fatty Acids (FAs) | Arachidonic acid (AA) |
|  |  | Docosahexaenoic acid (DHA) |
|  |  | Eicosapentaenoic acid (EPA) |
|  |  | Linoleic acid (LA) |
|  |  | Linolenic acid (LnA) |
|  |  | Oleic acid (OA) |
|  |  | Palmitic acid (PA) |
|  |  | Stearic acid (SA) |
| Monoglycerides | 2-Monoacyl glycerols (2-MAGs) | 2&1-Arachidonoyl glycerol (2-AG) |
|  |  | 2&1-Docosahexaenoyl glycerol (2-DHG) |
|  |  | 2&1-Linolenoyl glycerol (2-LnG) |
|  |  | 2&1-Oleoyl glycerol (2-OG) |
|  |  | 2&1-Palmitoyl glycerol (2-PG) |
|  |  | 2&1-Steareoyl glycerol (2-SG) |
| Fatty acid amides | N-acyl ethanolamides (N-EAs) (Fatty acid ethanolamides) | Arachidonoyl ethanolamide (AEA) |
|  |  | Docosahexaenoyl ethanolamide (DHEA) |
|  |  | Linoleoyl ethanolamide (LEA) |
|  |  | Oleoyl ethanolamide (OEA) |
|  |  | Palmitoyl ethanolamide (PEA) |
|  |  | Stearoyl ethanolamide (SEA) |
|  | N-acyl amides (N-Ams)  (Fatty acid primary amides) | N-Linolenoyl amide (Ln-Am) |
|  |  | N-Linoleoyl amide (L-Am) |
|  |  | N-Palmitoyl amide (P-Am) |
| N-Acyl Amino Acids | N-acyl serines (N-Sers) | N-Arachidonoyl serine (A-Ser) |
|  |  | N-Linoleoyl serine (L-Ser) |
|  |  | N-Oleoyl serine (O-Ser) |
|  |  | N-Palmitoyl serine (P-Ser) |
|  | N-acyl glycines (N-Glys) | N-Arachidonoyl glycine (A-Gly) |
|  |  | N-Docosahexaenoyl glycine (DH-Gly) |
|  |  | N-Linoleoyl glycine (L-Gly) |
|  |  | N-Oleoyl glycine (O-Gly) |
|  |  | N-Palmitoyl glycine (P-Gly) |
|  |  | N-Stearidonoyl glycine (S-Gly) |
|  | N-acyl alanines (N-Alas) | N-Linoleoyl alanine (L-Ala) |
|  |  | N-Oleoyl alanine (O-Ala) |
|  |  | N-Palmitoyl alanine (P-Ala) |
|  | N-acyl leucines (N-Leus) | N-Arachidonoyl leucine (A-Leu) |
|  |  | N-Docosahexaenoyl leucine (DH-Leu) |
|  |  | N-Linoleoyl leucine (L-Leu) |
|  |  | N-Oleoyl leucine (O-Leu) |
|  |  | N-Palmitoyl leucine (P-Leu) |
|  | N-acyl valines (N-Vals) | N-Linoleoyl valine (L-Val) |
|  |  | N-Oleoyl valine (O-Val) |
|  | N-acyl phenylalanines (N-Phes) | N-Linoleoyl phenylalanine (L-Phe) |
