# Supplementary material for: The association of circulating endocannabinoids with neuroimaging and blood biomarkers of neuro-injury
Source: Alzheimers Res Ther. 2023 Sep 12;15:154. doi: 10.1186/s13195-023-01301-x (PMC10496329; doi:10.1186/s13195-023-01301-x)
Supplement: Supplementary file 9 — Additional file 9: Supplementary Table 6. Summary of the study results. [file 13195_2023_1301_MOESM9_ESM.docx]

**Supplementary Table 6.** Summary of the study results

| Differences in endocannabinoid levels between women and men | | | | |
| --- | --- | --- | --- | --- |
| Fatty Acids | LA | Levels are significantly higher in women compared to men after correction for multiple testing | | |
|  | LnA |  |  |  |
| Fatty acid amides | OEA |  |  |  |
| N-Acyl Amino Acids | P-ser | Levels are significantly higher in men compared to women after correction for multiple testing | | |
|  | L-Leu |  |  |  |
|  | O-Leu |  |  |  |
|  | P-Leu |  |  |  |
|  | A-Leu |  |  |  |
|  | L-Val |  |  |  |
| Total sample | | | | |
|  | No significant associations with all outcomes after correction for multiple testing | | | |
| Samples stratified by sex ^a^ | | | | |
| Fatty Acids | AA | Gray matter | Women | n.s |
|  |  |  | Men | n.s |
|  |  | GFAP | Women | Negative (n.s) |
|  |  |  | Men | Negative |
|  | DHA | GFAP | Women | Negative (n.s) |
|  |  |  | Men | Negative |
|  | EPA | Total tau | Women | n.s |
|  |  |  | Men | n.s |
|  |  | UCH-L1 | Women | n.s |
|  |  |  | Men | n.s |
|  | LA | Hippocampal | Women | n.s |
|  |  |  | Men | n.s |
|  | LnA | NfL | Women | n.s |
|  |  |  | Men | n.s |
|  |  | GFAP | Women | Negative (n.s) |
|  |  |  | Men | Negative |
|  | PA | Hippocampal | Women | Positive |
|  |  |  | Men | Negative (n.s) |
| Monoglycerides | 2-AG | Total tau | Women | n.s |
|  |  |  | Men | n.s |
|  | 2-LnG | Gray matter | Women | Negative (n.s) |
|  |  |  | Men | Positive |
|  | 2-OG | Total tau | Women | n.s |
|  |  |  | Men | n.s |
|  | 2-PG | Total cerebral brain | Women | n.s |
|  |  |  | Men | n.s |
|  | 2-SG | NfL | Women | n.s |
|  |  |  | Men | n.s |
|  |  | Total tau | Women | n.s |
|  |  |  | Men | n.s |
| Fatty acid amides | AEA | Gray matter | Women | n.s |
|  |  |  | Men | n.s |
|  |  | Hippocampal | Women | n.s |
|  |  |  | Men | n.s |
|  |  | Total tau | Women | n.s |
|  |  |  | Men | n.s |
|  | DHEA | Total tau | Women | n.s |
|  |  |  | Men | n.s |
|  |  | UCH-L1 | Women | n.s |
|  |  |  | Men | n.s |
|  | LEA | Hippocampal | Women | Positive |
|  |  |  | Men | Negative |
|  |  | WMH | Women | n.s |
|  |  |  | Men | n.s |
|  |  | NfL | Women | n.s |
|  |  |  | Men | n.s |
|  |  | GFAP | Women | n.s |
|  |  |  | Men | n.s |
|  | OEA | Hippocampal | Women | n.s |
|  |  |  | Men | n.s |
|  | PEA | Hippocampal | Women | n.s |
|  |  |  | Men | n.s |
|  | P-Am | GFAP | Women | n.s |
|  |  |  | Men | n.s |
|  |  | Total tau | Women | n.s |
|  |  |  | Men | n.s |
| N-Acyl Amino Acids | A-Ser | Gray matter | Women | n.s |
|  |  |  | Men | n.s |
|  |  | Hippocampal | Women | n.s |
|  |  |  | Men | n.s |
|  | L-Ser | Gray matter | Women | n.s |
|  |  |  | Men | n.s |
|  |  | WMH | Women | Positive (n.s) |
|  |  |  | Men | Negative |
|  | P-Ser | NfL | Women | n.s |
|  |  |  | Men | n.s |
|  |  | GFAP | Women | Positive (n.s) |
|  |  |  | Men | Negative |
|  | L-Gly | Hippocampal | Women | n.s |
|  |  |  | Men | n.s |
|  |  | Total tau | Women | n.s |
|  |  |  | Men | n.s |
|  | O-Gly | Hippocampal | Women | n.s |
|  |  |  | Men | n.s |
|  | P-Gly | Hippocampal | Women | Positive |
|  |  |  | Men | Negative (n.s) |
|  | S-Gly | Total tau | Women | n.s |
|  |  |  | Men | n.s |
|  | L-Ala | Total tau | Women | n.s |
|  |  |  | Men | n.s |
|  | P-Ala | Hippocampal | Women | Positive |
|  |  |  | Men | Negative (n.s) |
|  |  | UCH-L1 | Women | n.s |
|  |  |  | Men | n.s |
|  | A-Leu | GFAP | Women | n.s |
|  |  |  | Men | n.s |
|  | L-Leu | Total tau | Women | n.s |
|  |  |  | Men | n.s |
|  | O-Leu | NfL | Women | Negative |
|  |  |  | Men | Negative (n.s) |
|  |  | Total tau | Women | n.s |
|  |  |  | Men | n.s |
|  | L-Val | NfL | Women | n.s |
|  |  |  | Men | n.s |
|  | O-Val | Total tau | Women | n.s |
|  |  |  | Men | n.s |
|  | L-Phe | Hippocampal | Women | Negative |
|  |  |  | Men | Positive (n.s) |
|  |  | Total tau | Women | n.s |
|  |  |  | Men | n.s |

Abbreviations: WMH=White matter hyperintensities; NfL= Neurofilament Light; GFAP= Glial Fibrillary Acidic Protein; UCH-L1=Ubiquitin Carboxyl-Terminal Hydrolase L1; n.s=non-significant; Positive=higher levels of the endocannabinoids are associated with higher levels of the outcome;

Negative=higher levels of the endocannabinoids are associated with lower levels of the outcome.

^a^ Sex stratification is shown only for models with significant eCBs*sex interaction.
